# A Study on the Morphometric, Macroanatomical Structure, and Arterial Vascularisation of the Upper Digestive System in Rabbits (*Oryctolagus cuniculus*, Linnaeus 1758)

**DOI:** 10.1002/vms3.70505

**Published:** 2025-07-11

**Authors:** Gülseren Kirbaş Doğan, Elif Duman Çabakçor

**Affiliations:** ^1^ Department of Anatomy Faculty of Veterinary Medicine Kafkas University Kars Turkey

**Keywords:** anatomy, digestive system, rabbit, X‐ray

## Abstract

Rabbits are widely used in biomedical and veterinary research due to some physiological and anatomical similarities to humans. Furthermore, their controllable breeding requirements make them suitable for laboratory experiments. Ten male rabbits were used in the presented study. The arteries supplying the upper digestive system organs were dissected. Morphometric and macroanatomical findings of these organs were taken and organs were imaged using X‐ray. In rabbits, the upper lip was found to have a cleft (philtrum). The number of palatine rugae on the palate was determined to be 13 pairs. The average linguae length in rabbits was measured as 52.39±1.75 mm. Torus of the tongue and median sulcus of the tongue was seen prominently on the tongue. Four types of papillae were macroanatomically detected on the linguae. These were filiform papillae, fungiform papillae, vallate papillae, and foliate papillae. The tongue muscles and arteries were dissected and named. The parotid gland was 28.4 mm in length, 57.245 mm in width, and 44.58 g in weight. Total oesophagus length was determined as 15.85 cm and weight was 2.41 g.

As a result, this study contributed to the anatomy literature on rabbit upper digestive system organs. In addition, the detailed data obtained will form the basis for future studies and operations on the rabbit upper digestive system.

## Graphical abstract

The average diastema length in the upper jaw was measured as 29.79 mm, while it was measured as 24.23 mm in the lower jaw. The average linguae length in New Zealand rabbits was measured as 52.39 ± 1.75 mm. The parotid gland was determined the largest length (28.4 mm), width (57.245 mm), and weight (44.58 g). Total oesophagus length was determined as 15.85 cm and weight was 2.41 g. The total oesophagus length was determined as 15.85 mm and weight as 2.41 g

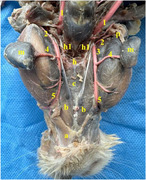

## 1 Introduction

Rabbits, which are in the *Leporidae* family, are important as laboratory animals due to their easy availability and the benefits they provide in research. They are also used for their meat. Rabbit breeding is widespread due to the fact that they can produce many offspring in a short time, are easy to care for, and can be used as food (Gültiken 2010). The nutrients and energy required for the survival of the living being, growth, and repair of worn‐out cells and tissues are obtained as a result of the digestion of the food consumed. The food substances consumed undergo changes in the digestive tract due to mechanical, enzymatic, chemical, and bacterial effects. The secretions of the pancreas, liver (hepar), stomach (gaster), and small intestines (intestinum tenue) are also very important as secretion factors in the transformation of nutrients in the digestive tract (König and Liebich 2015; Reece 2015). In order for nutrients to be used by cells and organs, they must be broken down to the extent that they can mix with the blood and lymph as a result of mechanical and chemical digestion (Dyce et al. 2010). The main goal of the digestive system organs is to break down the nutrients consumed into their building blocks and make them absorbable from the intestines (Demiraslan and Dayan 2021; Dyce et al. 2010).

The first part of the upper digestive system is the lips (labia). The lips are responsible for capturing food and transferring to the oral cavity (cavum oris). The tongue (lingua) is responsible for mixing food (Dyce et al. 2010). The saliva secreted by the salivary glands (glandulae salivariae) helps to create lubrication in the mouth and to moisten and soften the food taken in. The esophagus (oesophagus) is a tube‐shaped organ that allows food to pass from the pharynx to the stomach. Food coming from the esophagus is stored in the stomach, softened and chemically digested by the digestive enzymes produced (Reece 2015). The intestines (intestinum) are the part of the digestive tract that starts from the stomach and ends at the anus. The best absorption of digested food from the intestines depends on the size of the inner surface of the intestine. The larger the inner surface of the intestine, the better the absorption. The absorption surface is increased by the glove‐like mucosa called villi, which lines the inner surface of the intestine (Dursun 2012).

There are also significant differences in the structure and function of the digestive system among herbivores. When these differences are classified in general, they can be divided into three different classes. The first class consists of ruminants such as camelids, which have fermentation activity before digestion (Reece 2015). The second class includes posterior fermenters, such as horses and rodents, which have a large intestine (caecum and colon) as a fermentation cycle. The third class includes caecotrophs, such as rabbits and hares, which named caecotrophs (Cotozzolo et al. 2021).

Rabbits have a digestive system that allows for high food intake and therefore high energy and protein intake. The digestive organs are responsible for the removal of digestible and easily fermentable components of the diet and for the rapid removal and transportation of fibrous waste that would otherwise ferment slowly (Cotozzolo et al. 2021). The contents are completely separated in the large intestine (intestinum crassum) and distributed over a large absorptive surface area. Rabbits have a mechanism that allows the caecal fermentation products and feces to be re‐ingestioned and bacteria to absorb their by‐products from the small intestine (Davies and Davies 2003).

In rabbits, lower jaw teeth (mandibular teeth) grow faster than upper jaw teeth (maxillary teeth). For this reason, hard foods are given to wear down the teeth (Killman 2009). The teeth formula of the rabbit is I 2/1, C 0/0, PM 3/2, M 3/3 and there are 28 teeth in total. As in all lagomorphs, rabbits have three pairs of incisor teeth, two in the upper jaw bone (maxilla) and one pair in the lower jaw bone (mandibula) (Craigie 1969). The second incisor teeth in the upper jaw bone is underdeveloped. There is no canin teeth in the toothless area known as diastema between the incisor teeth and premolar teeth (Killman 2009). If the diet does not contain enough hard material, irregular extensions may form as the teeth cannot wear properly. This can lead to chewing problems such as malocclusion. Malocclusion can be prevented by including plenty of good quality dry grass in the diet (Craigie 1969, Gültiken 2010).

There are studies on the digestive system in different laboratory animals, such as Balb‐c and Swiss albino mouse (Başdinç, 2023), Balb‐c and Swiss albino mouse (Malewitz 1965), mouse, (Cooper and Schiller 1975), guinea pig (Potter et al.1956), rats (Greene 1963) and (Vdoviaková et al. 2016). However, it was determined that the studies conducted on rabbits were limited. Therefore, it was planned to examine the morphometric and macroanatomical structures of the upper digestive system organs morphometry, macroanatomy, and the arteries supplying these organs in detail in the rabbit. At the same time, organs were tried to be shown using imaging methods such as ultrasound and X‐ray.

The aim of the presented study will contribute to the veterinary anatomy literature by examining the macroanatomical structure and morphometric values of the upper digestive system organs in the rabbit in detail and determining the arteries that feed these organs. It was also aimed to help clinicians by determining the locations and images of the upper digestive system organs with X‐ray techniques.

## 2 Materials and Methods

### 2.1 Research Permission

An application was made to Kafkas University Animal Experiments Local Ethics Committee (…‐HADYEK) for permission for this study. Our study was approved with the code KAU‐HADYEK/2023‐083.

### 2.2 Animal Material

**FIGURE 1:**
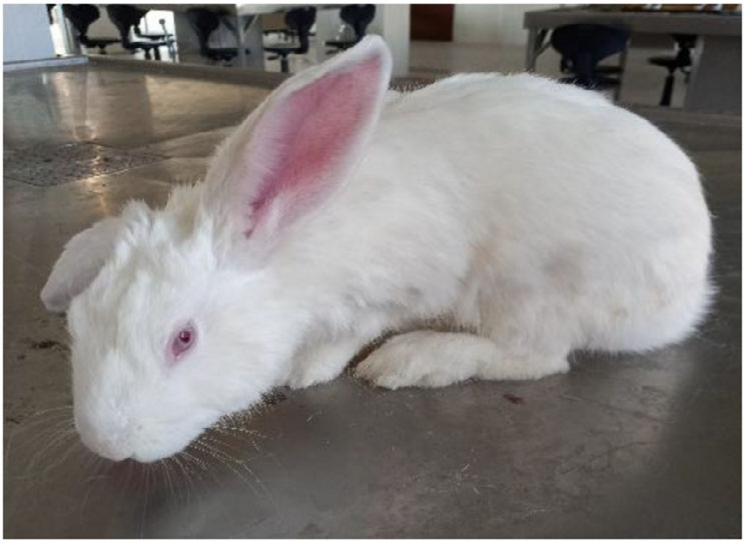
New Zealand Rabbit used in the presented study.

Materials obtained from Kafkas University Medical Experimental Application and Research Center, which has an official document for breeding and selling experimental animals (Figure 1), were brought to Kafkas University Veterinary Faculty Anatomy Department laboratory and the study was conducted. In this study 10 male, 7 months old, with an average weight of 2862.10 ±73.90 g animals were used rabbits. Firstly after the anaesthesia procedure, two rabbits were examined with X‐ray. After the imaging process was completed, the anesthetised animals were drained of blood. Latex was injected into two of the rabbits to determine the arteries. The findings from eight rabbits were evaluated for morphological and macroanatomical findings.

### 2.3 Method

Animal materials were anesthetised intramuscularly (i.m.) with a combination of 5 mg Xylazin HCL (rompun) and 35 mg Ketamine (Ketalar) (Holmes 1988). The materials were transported to the Anatomy Department Laboratory of the Faculty of Veterinary Medicine at Kafkas University and the procedures to be applied were carried out here. Radiological findings of the upper digestive system of the anesthetised animals were performed with ORINE IM 82 (50Kvp–10mA 500VA XRAY Tube: CEI OCX 50) brand X‐ray device and Fujifilm FCR PRIMA T2 brand computerised X‐ray device. After these procedures, the common carotid artery (arteria carotis communis) of the anesthetised rabbits were cut and the blood was drained. The dissection procedure was started by considering the applications in Mukhopadhyay and Wagner (2020) and Perpiñán (2019). These applications consist of seven incisions that aim to reach the internal organs of the animal without damaging them. The first incision was made along the median line without damaging the diaphragm. The second incision was made as a transverse incision from the ventral edge of the diaphragm along the lateral left and right lines. The third incision was made parallel to the second incision, forming an arch over the diaphragm. The fourth incision was completed by placing the scalpel in the tissue at the midline of the sternum and continuing along the median line to the lower jaw bone. The fifth incision was made as a transverse incision laterally in both directions, left and right, along the dorsal edge of the pectoralis major muscle (musculus pectoralis major). The sixth incision was continued from the ventral side of the diaphragm arch to the pelvis along the median line. The seventh incision was made as a lateral left and right incision from the most ventral point of the median line incision. After the dissected organs were photographed, the length, width, and thickness of each organ were measured with a digital caliper. Finally, the organ sections were separated and each was weighed on a precision scale (min 0.0001 g, max 220 g, precisa code XB220A). Measurements were taken while the lumen organs were full. The measured morphometric values were evaluated in the Statistical Package for the Social Sciences (SPSS) 20 program. Latex application was performed on two rabbits whose vessels were washed with physiological serum to reveal the arteries that vascularise the upper digestive system organs. Latex (ZPK‐580‐S; Gerard Biological Center, Preston UK) coloured with red fabric dye (Artdeco) was injected into the right common carotid artery (arteria carotis communis dextra) and the left common carotid artery (arteria carotis communis sinistra) dissected from the neck area, ensuring that all arteries were filled with this mixture (Bugge 1963; Erençin et al. 1967; Aycan and Bilge 1984). After the the right common carotid artery and the left common carotid artery were ligated, the materials were kept at room temperature for 24 h for the latex in the materials to freeze. After it was understood that the latex had frozen, the materials were stored in 10% formalin solution for a week. After all formations were fixed in formalin, the materials were dissected. The other eight animals were fixed in formalin solution after euthanasia. The organs were weighed step by step, their lengths were measured and macroanatomical findings were obtained. In order to determine the location of the digestive system organs vertebralally, a dorsal vertebral dissection of a material preserved in formalin was performed. In order to verify these findings, macroanatomical and morphometric findings were compared and supported with X‐ray. The pictures were taken with an iPhone 15 brand phone camera. Nomina Anatomica Veterinaria (N.A.V. 2017) was used for naming anatomical terms.

### 2.4 Statistical Analysis

Data from all measurements taken on upper digestive system organs in animals were evaluated using the SPSS (20.0 version) package program. Descriptive statistics such as mean, standard deviation, median, minimum‐maximum values ​​were used for numerical parameters.

## 3 Results

### 3.1 Cavum Oris (Oral Cavity)

**FIGURE 2:**
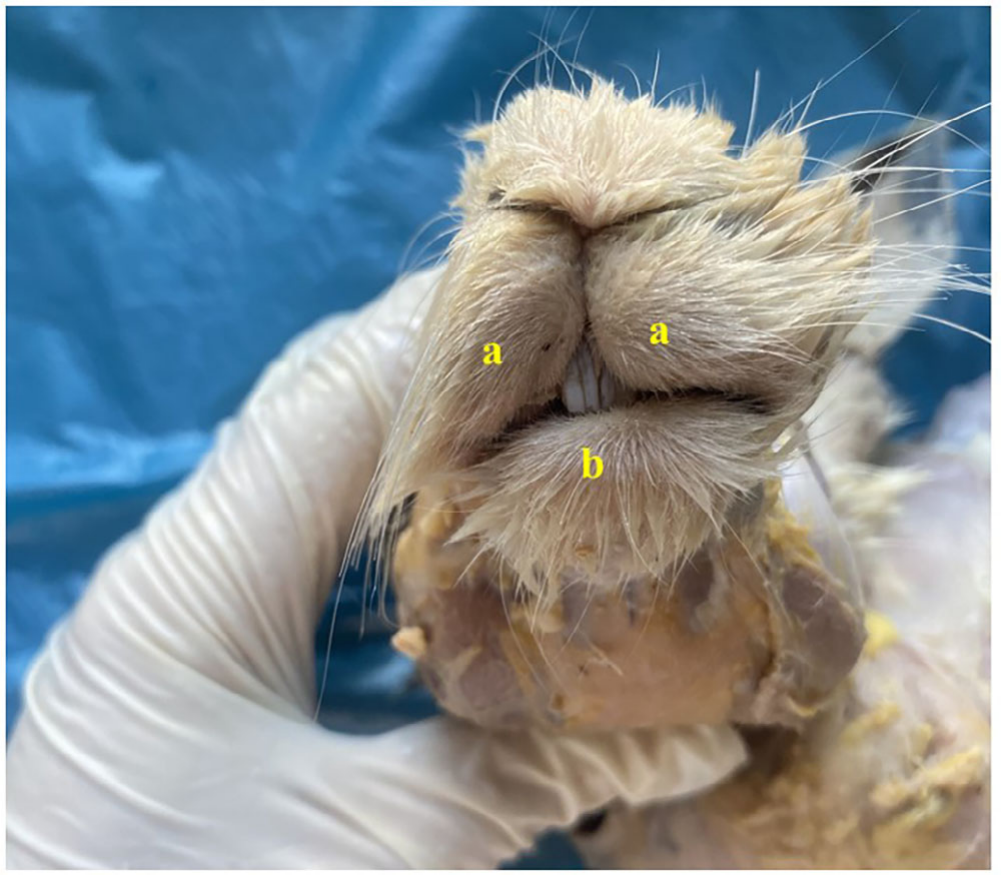
Labia oris in New Zealand rabbits (**a**: upper lip **b**: lower lip).

It was seen that the rima oris, which is the cleft between the upper lip (labium superius‐maxillaris) and lower lip (labium inferius‐mandibularis), was narrow (Figure 2). It was appeared that the main oral cavity (cavum oris proprium), which remained within the tooth arches, was larger than the vestibulum oris. It was revealed that the connection between the main oral cavity and the buccal cavity (vestibulum oris) was provided by the diastema due to the absence of canine teeth when the teeth in the lower and upper jaws were united.

### 3.2 Labia Oris (Lips)

**FIGURE 3:**
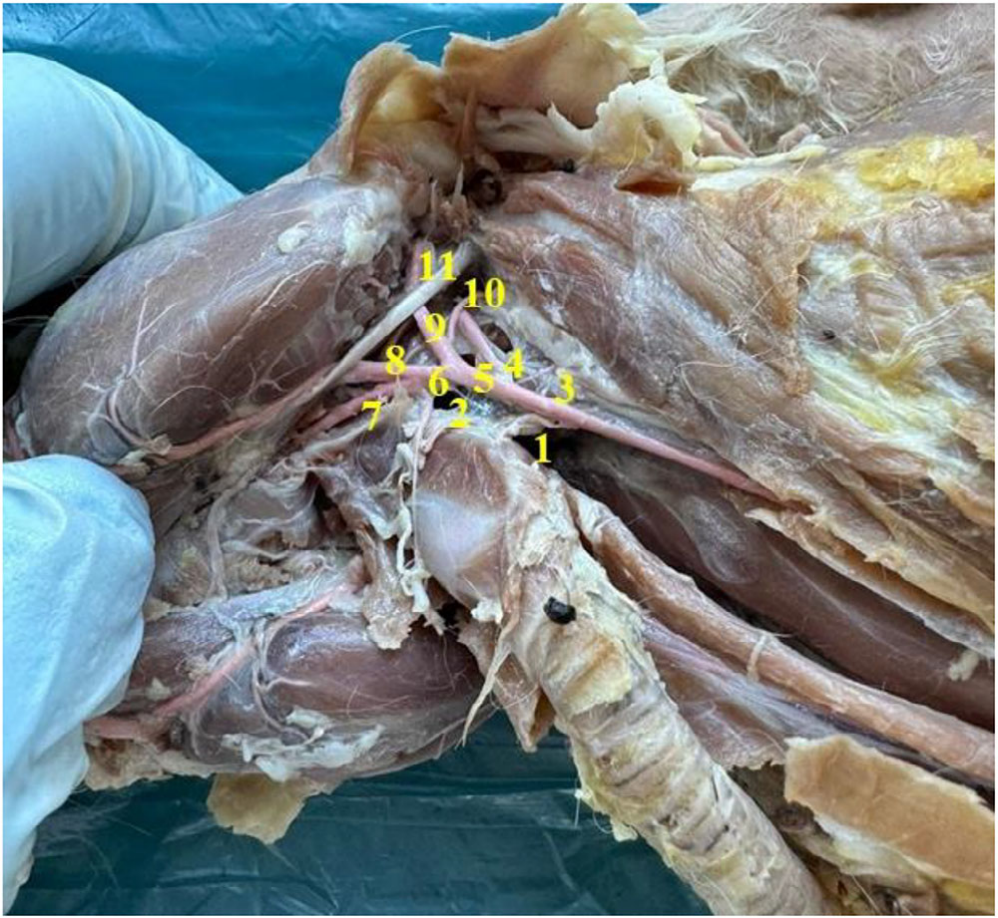
Common carotid artery and its branches in rabbits (**1**: ramus thyroideus (a. thyroidea cranialis), **2**: thyroid gland, **3**: commun carotid artery, **4**: internal carotid artery, **5**: external carotid artery, **6**: linguofacial trunk, **7**: lingual artery, **8**: facial artery, **9**: superficial temporal artery, **10**: occipital artery, **11**: tendon of musculus geniohyoideus).

**FIGURE 4:**
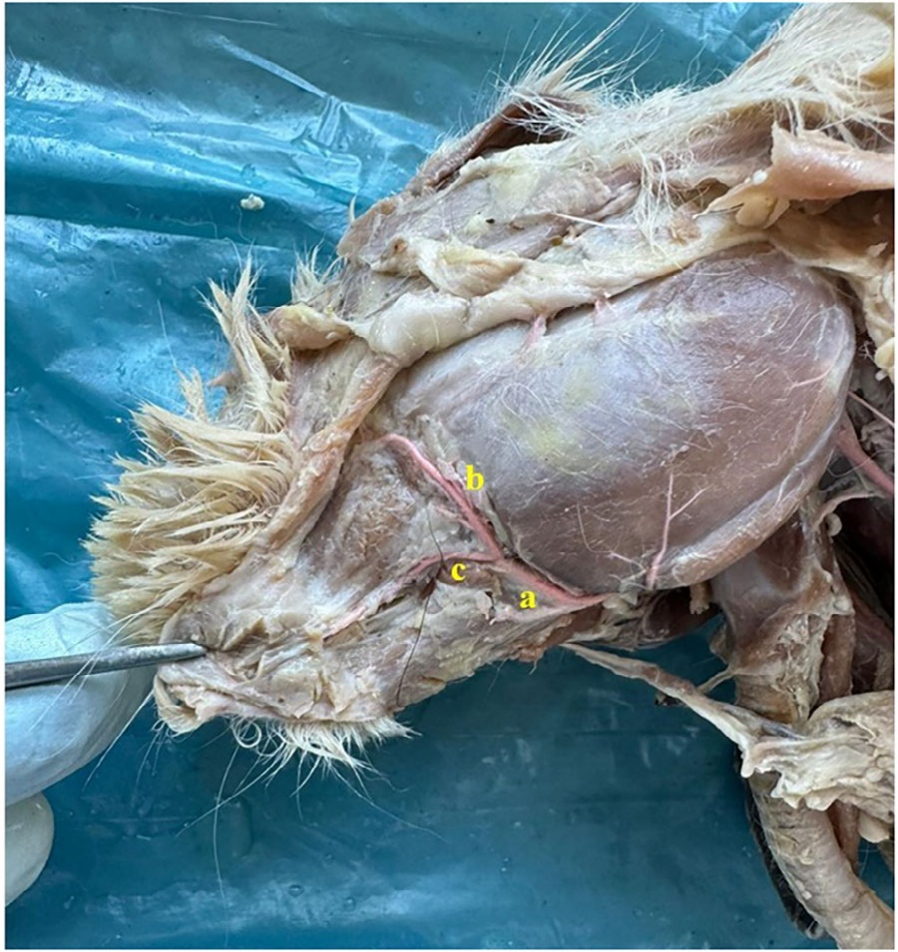
Arterial vascularisation of the labia in rabbits (**a**: facial artery, **b**: superior labial artery, **c**: inferior labial artery).

In rabbits, the upper lip was found to have a cleft (philtrum) (Figure 2). It was determined that the inferior labial artery (arteria labialis inferior), which originates from the facial artery, was distributed in the lower lip. It was appeared that the superior labial artery (arteria labialis superior), which is a strong branch that continues from the facial artery (arteria facialis), vascularises the the upper lip (Figures 3 and 4).

### 3.3 Dentes (Teeth)

**FIGURE 5:**
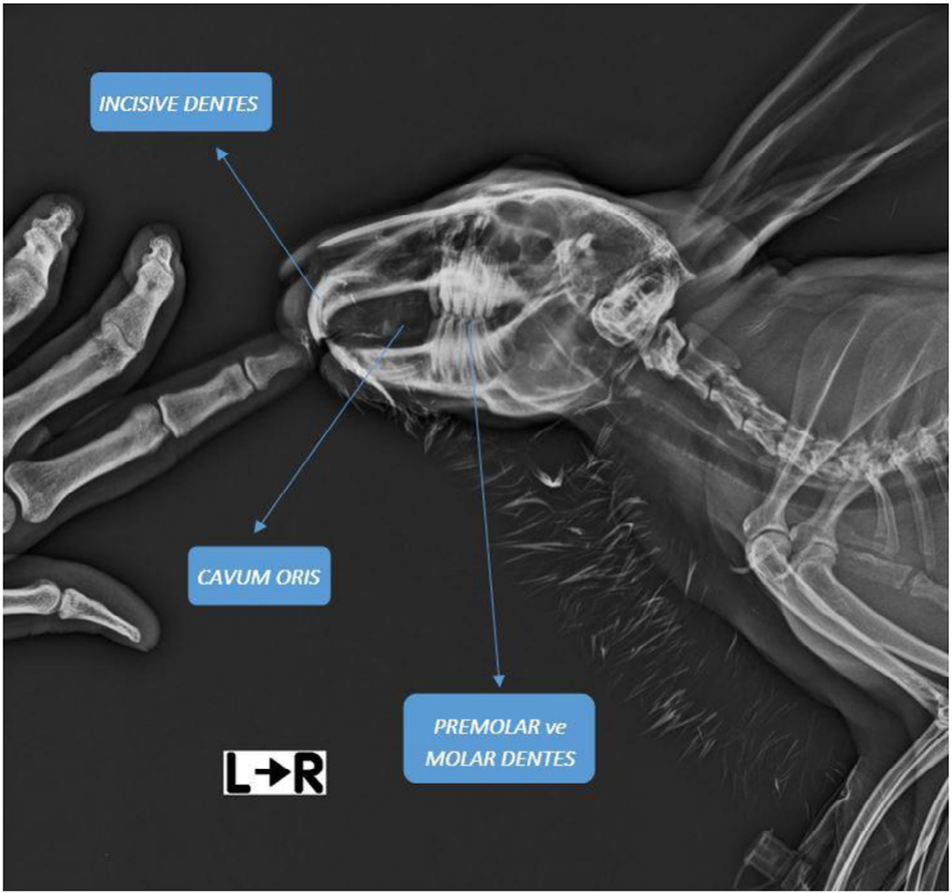
X‐ray image of oral cavity and teeth in rabbits.

It was seen that rabbits had 2 incisors in the upper jaw, 1 incisor in the lower jaw, 3 premolars in the upper jaw, 2 premolars in the lower jaw, 3 molars in the upper jaw, and 3 molars in the lower jaw. It was revealed that the first and second incisors in the upper jaw bone were of similar size. It was appeared that there were two secondary upper jaw incisors immediately caudal to the lower jaw incisors in the upper jaw. It was determined that there were no canine teeth in the toothless area known as diastema between the incisors and premolars in both the upper and lower jaws. The average diastema length in the upper jaw was measured as 29.79 mm, while it was measured as 24.23 mm in the lower jaw. The X‐ray image of the oral cavity and teeth in rabbits is shown in Figure 5. Parameters of the teeth are presented in Table 1.

**TABLE 1:** Teeth morphometric parameters (dentes) (mm).

	Parameters	Minimum	Maximum	Mean + standard deviation
**Half of the upper jaw**	Total incisive tooth surface length	2.00	2.78	2.39±0.28
	Total premolar tooth surface length	3.00	3.44	3.21±0.16
	Total molar tooth surface length	3.00	3.52	3.27±0.16
	Width of incisive teeth	3.12	3.41	3.28±0.08
	Length of incisive teeth	5.59	8.32	7.08±0.91
	Distance between incisors and premolars	28.46	31.00	29.79±0.85
	The distance between the beginning of the premolar teeth and the end of the molar teeth	14.44	16.48	15.16±0.71
**Half of the lower jaw**	Total incisive tooth surface length	1.00	1.43	1.18±0.13
	Total premolar tooth surface length	3.00	3.29	3.18±0.10
	Total molar tooth surface length	2.04	2.41	2.21±0.12
	Width of incisive teeth	3.09	3.47	3.28±0.13
	Length of incisive teeth	5.53	7.07	6.36±0.53
	Distance between incisors and premolars	23.54	25.98	24.23±0.80
	The distance between the beginning of the premolar teeth and the end of the molar teeth	12.30	19.66	15.68±2.17

### 3.4 Palatum (Palate)

**FIGURE 6:**
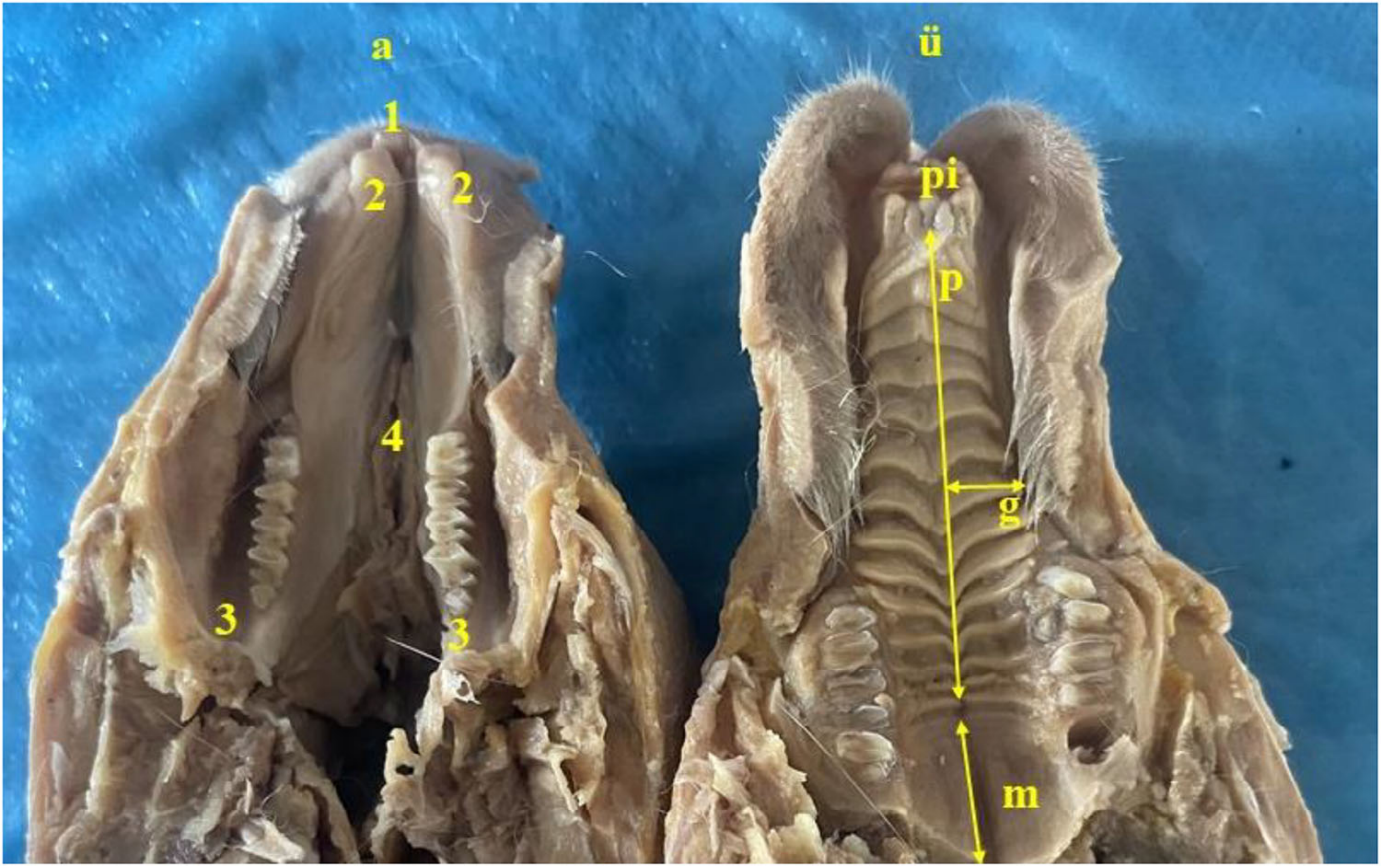
Anatomical structures seen in the oral cavity of rabbits (**a**: lower jaw, **ü**: upper jaw, **1**: mandibular incisiv teeth, **2**: mucosal extension of the lower cheek, **3**: buccopharyngeal cavity, **4**: area where frenulum of the tongue is attached, **pi**: incisiv papilla, **p**: hard palate, palatine raphe, **g**: palatine rugae, **m**: soft palate).

It was seen that the hard palate (palatum durum) was formed by transverse processes called palatine rugae (rugae palatinae) and the palatine raphe (raphe palatina) formed by the symmetrical union of these processes on the median line. The number of palatine rugae was determined to be 13 pairs. It was appeared that eight pairs of palatine rugae were located in the diastema area. It was revealed that the hard palate located in the cranial area had a rougher structure while the soft palate (palatum molle) located in the caudal area had a softer and smoother structure. The patency of the nasopalatinal canal (canalis nasopalatinus), which is found in pairs in the cranial area of the hard palate, was appeared. In the present study, it was seen that the ascending palatine artery (arteria palatina ascendens), which starts from the maxillary artery (arteria maxillaris), provided the vascularisation of the hard palate. It was determined that the ascending palatine artery and the major palatine artery (arteria palatina major) provided the arterial vascularisation of the small soft palate. Anatomical structures in the oral cavity are shown in Figure 6. Data for the palatum are presented in Table 2.

**TABLE 2:** Palate parameters (mm).

Parameters	Minimum	Maximum	Mean + standard deviation
**RP**	32.90	36.41	34.43±1.11
**RPE**	14.04	16.00	15.18±0.78
**RPB**	13.00	14.67	13.77±0.64

Abbreviations: RPB, length of rugae palati; RPE, width of rugae palati; RP, length of raphe palate.

### 3.5 Lingua (Tongue)

**FIGURE 7:**
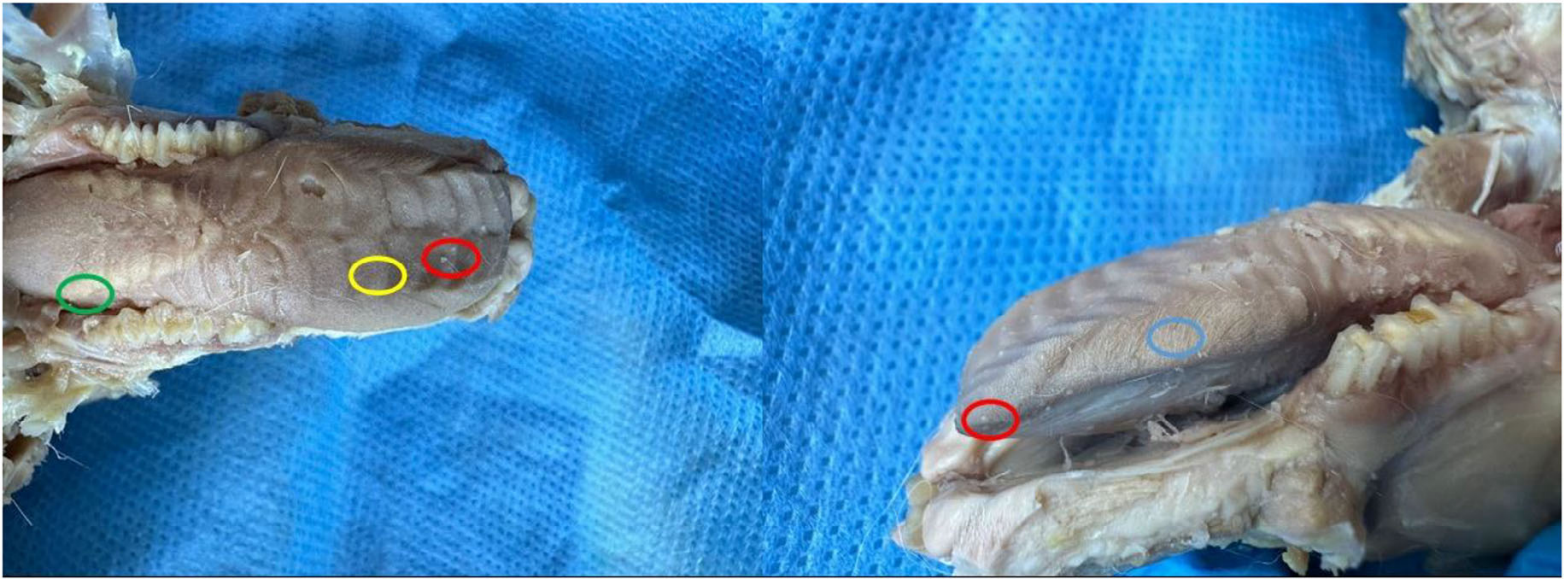
Papillae on the tonguae of rabbits (**yellow circle**: filiform papillae, **red circle**: fungiform papillae, **blue circle**: foliatae papillae, **green circle**: vallatae papillae).

**FIGURE 8:**
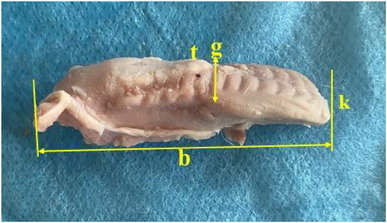
Measurements taken from tongue in the Rabbit (**b**: length of the tongue, **g**: width of the tongue, **k**: thickness of the tongue, **t**: torus of the tongue).

**FIGURE 9:**
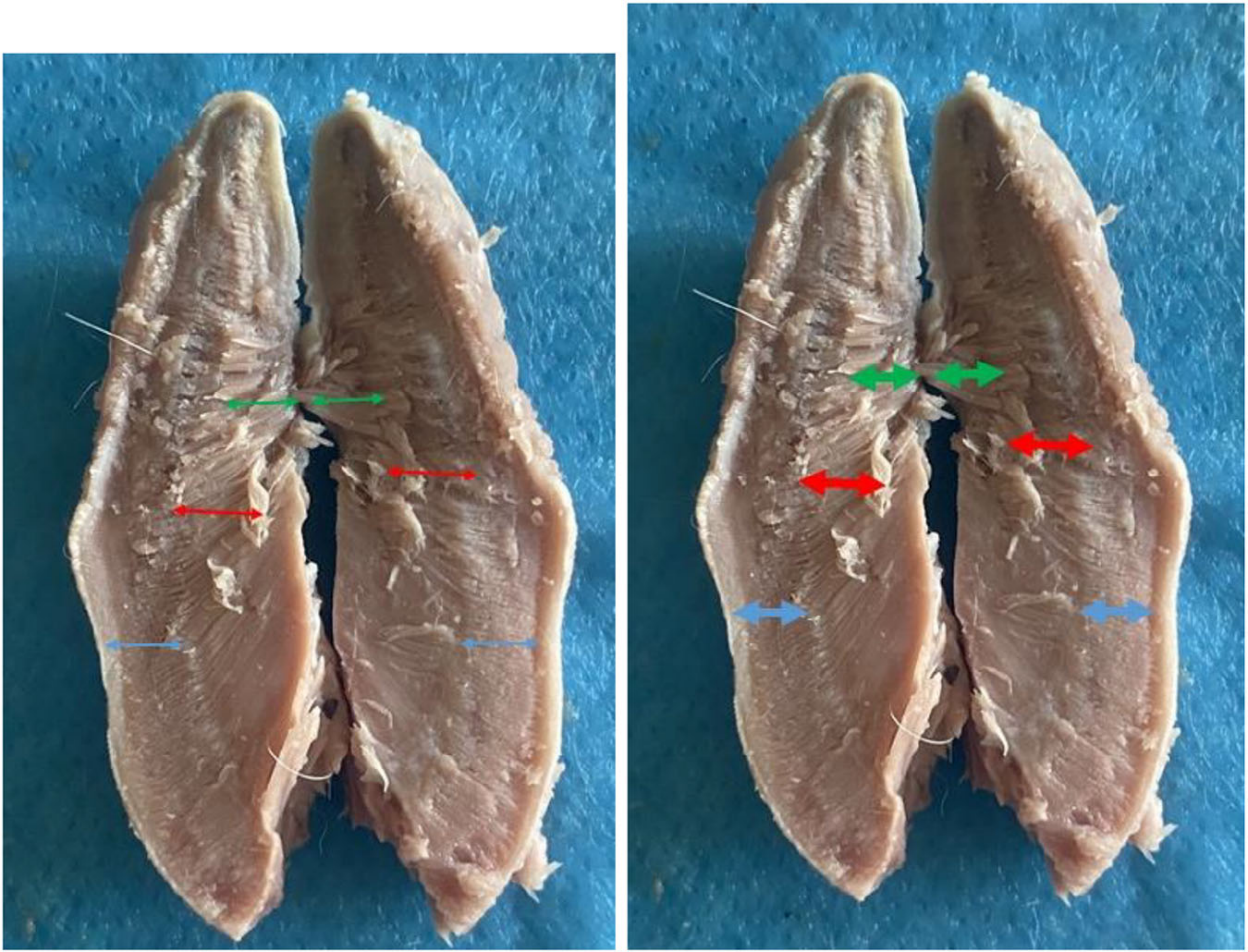
Lingual muscles seen symmetrically after the tongue is separated from the median line in the Rabbit (**blue arrow**: longitudinal and superficial muscle fibres located in the dorsal part (fibrae longitudinales superficiales), **red arrow**: The vertical and transverse muscle fibres located in the middle part (fibrae transversae ve fibrae perpendiculares), **green arrow**: Longitudinal and deep muscle fibres located in the ventral part (fibrae longitudinales profundae).

**FIGURE 10:**
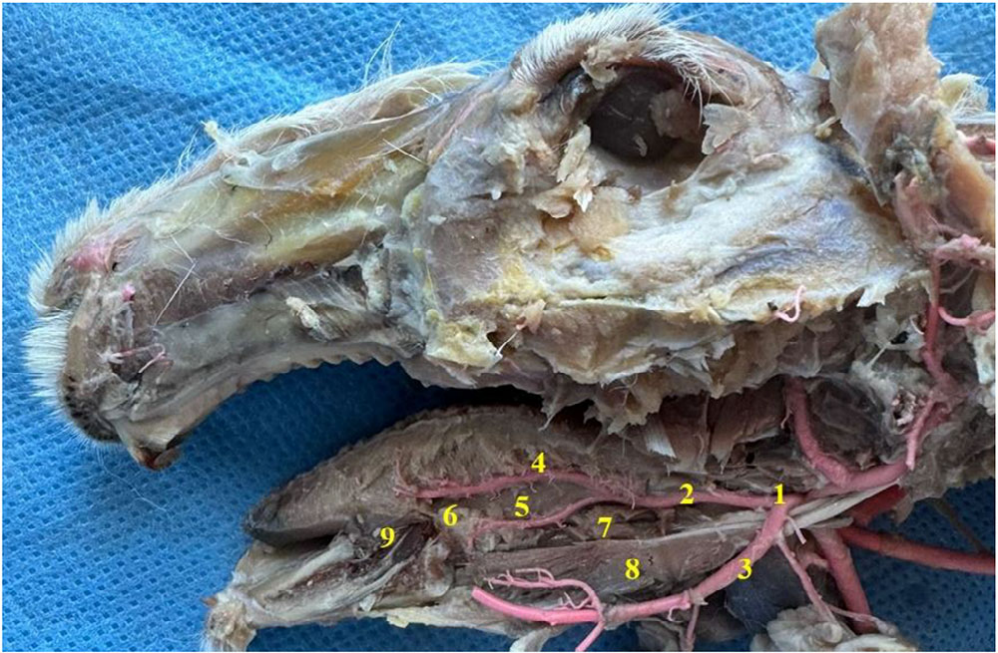
Arterial vascularisation of the tongue in the Rabbit and tongue muscles seen from the lateral view (**1**: linguofacial trunk, **2**: lingual artery, **3**: facial artery, **4**: deep linguistic artery, **5**: sublingual artery, **6**: sublingual gland, **7**: hyoglossus muscle, **8**: geniohyoideus muscle, **9**: genioglossus muscle).

Tongue was seen to be a muscular organ located inside the oral cavity. It was determined that it gradually rose towards the root of the tongue (radix linguae) and formed the torus of the tongue (torus linguae). It was observed that the median sulcus of the tongue in the median, which divided the linguae into symmetrical halves, extended from the tip of the tongue (apex linguae) of the tongue to the body of the tongue (corpus linguae) and ended in front of the torus of the tongue. Four types of papillae were macroanatomically detected on the tongue. It was determined that the filiform papillae (papillae filiformis) were numerous among the fungiform papillae on the dorsal, especially cranial, tip of the tongue. It was appeared that the fungiform papillae were papillae with mushroom‐shaped protrusions located along the dorso‐rostral edge of the tongue and even shifted towards the ventral of the tip of the tongue. Fungiform papillae were scattered sparsely among the filiform papillae, which were the most numerous. Vallate papillae were seen to be located especially on the root of the tongue and body of the tongue of the tongue. Foliatae papillae were found to be localised on the caudal and lateral sides of the tongue (Figure 7). The measurements taken on the tongue in the rabbit are shown in Figure 8. The muscles belonging to the tongue (musculus lingualis proprius), which are seen symmetrically after the tongue is separated from the median line, are shown in Figure 9. The length of the tongue was measured as 52.39 ± 1.75 mm. It was seen that the external muscles of the tongue are formed by mylohyoid muscle (musculus mylohyoideus), hyoglossus muscle (musculus hyoglossus), basioglossus muscle (musculus basioglossus), ceratoglossus muscle (musculus ceratoglossus), chondroglossus muscle (musculus chondroglossus), genioglossus muscle (musculus genioglossus), sternohyoideus muscle (musculus sternohyoideus), sternothyrohyoideus muscle (musculus sternothyrohyoideus), thyrohyoideus muscle (musculus thyrohyoideus), styloglossus muscle (musculus styloglossus). Tongue muscles and the arterial vascularisation of the tongue are shown in Figure 10. It was seen that the arterial vascularisation of the tongue is provided by the lingual artery (arteria lingualis), which originates from the linguofacial trunk (truncus linguofacialis) (Figures 3 and 10). It was determined that the lingual artery coursed cranial between the genioglossus and hyoglossus muscles. It was appeared that the lingual artery divided into two branches, the deep lingul artery (arteria lingualis profunda) and sublingual artery (arteria sublingualis), at the level of the root of the tongue (Figure 10). Parameters of the tongue are presented in Table 3.

**TABLE 3:** Parameters of the linguae (mm).

Parameters	Minimum	Maximum	Mean + standard deviation
**LİNB**	50.16	55.19	52.39±1.75
**LİNG**	13.49	20.25	17.38±2.20
**LİNK**	7.70	18.85	14.93±3.40
**LİNA**	4.08	7.95	5.82±1.13

Abbreviations: LİNB, length of the linguae; LİNG, width of the linguae; LİNK, thickness of the linguae; LİNA, weight of the linguae.

### 3.6 Glandulae Salivariae (Salivary Glands)

**FIGURE 11:**
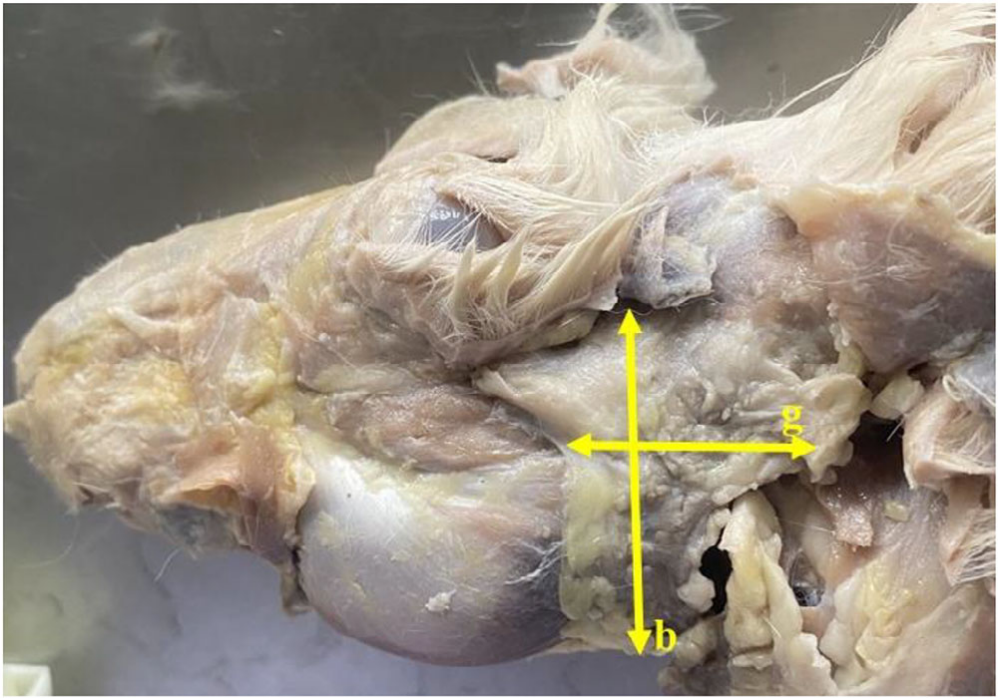
Parotid gland and measurements taken in the rabbit (**b**: length of the parotid gland, **g**: width of the parotid gland).

**FIGURE 12:**
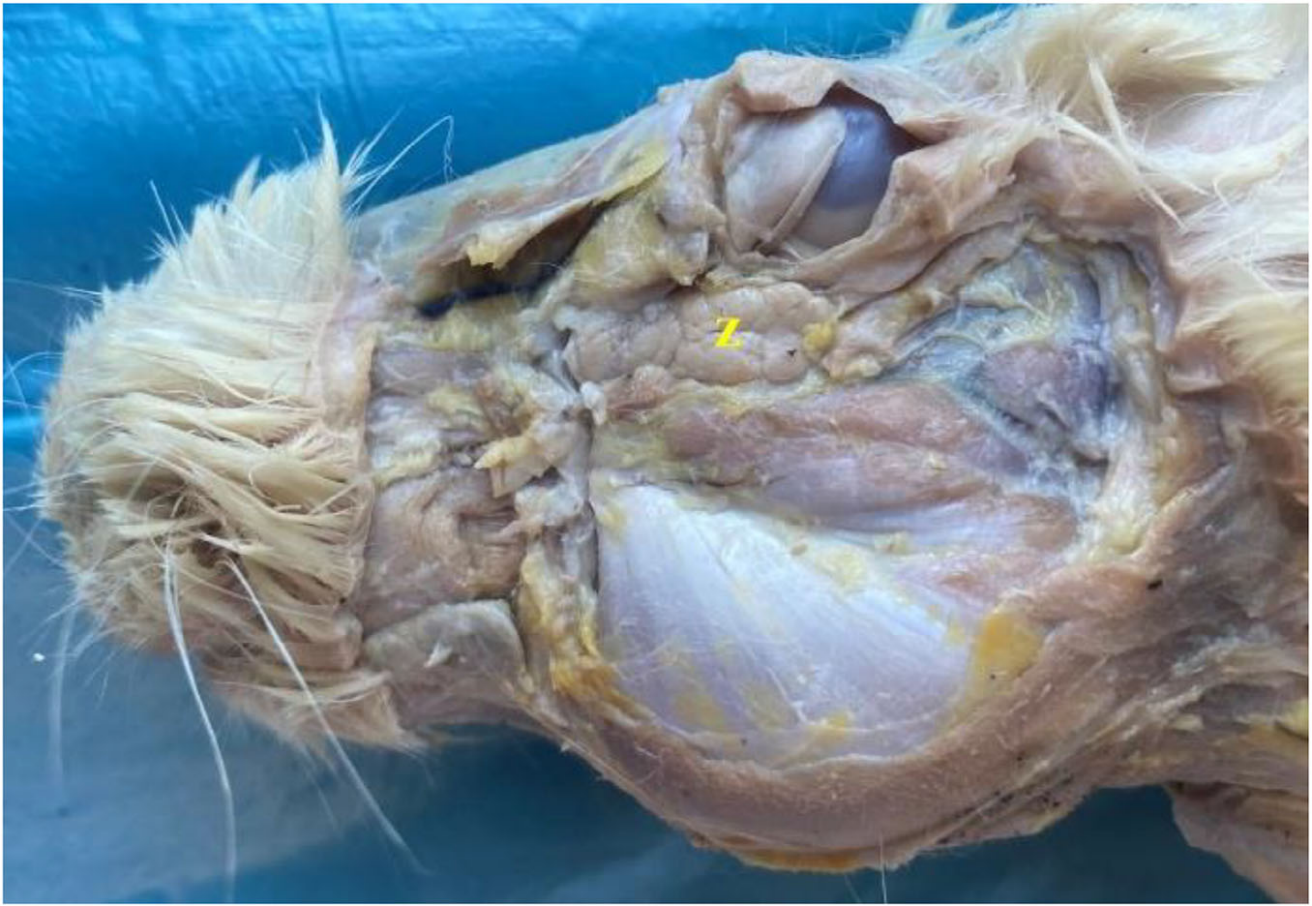
Zygomatic gland in the rabbit (**z**: zygomatic gland).

**FIGURE 13:**
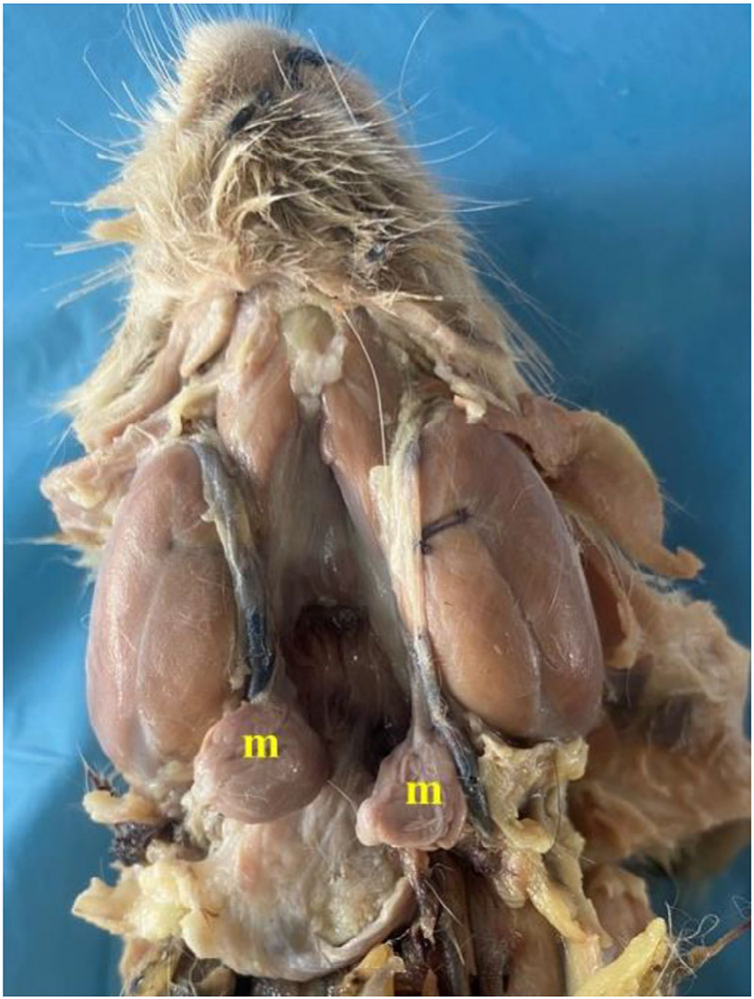
Mandibular gland in the rabbit (**m**: mandibular gland).

**FIGURE 14:**
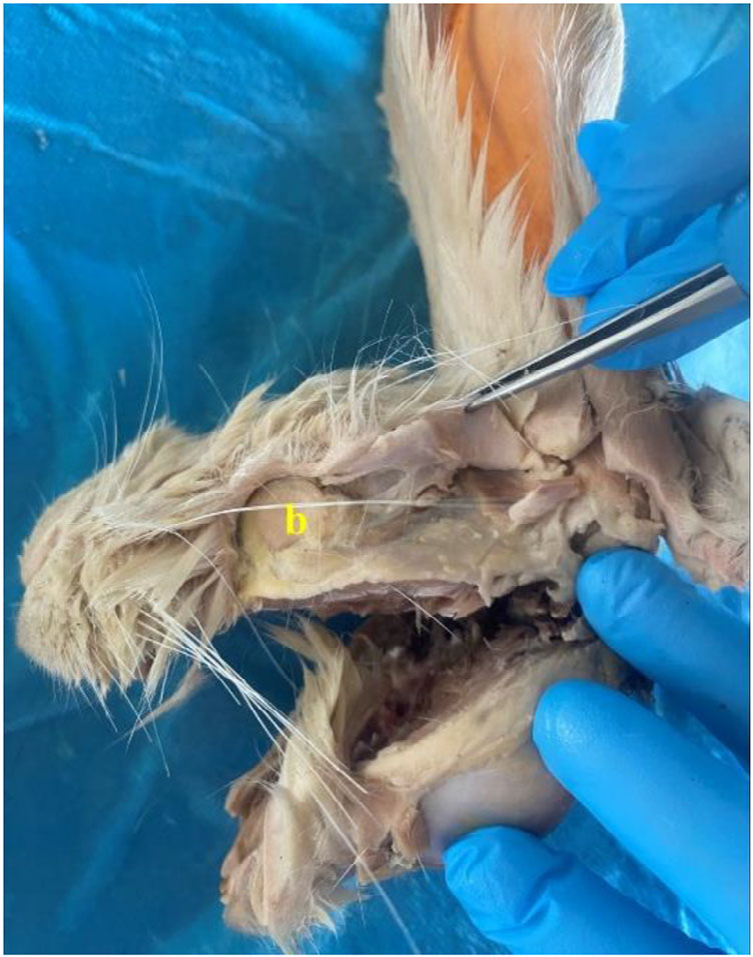
Buccal gland in the rabbit (**b**: buccal gland).

**FIGURE 15:**
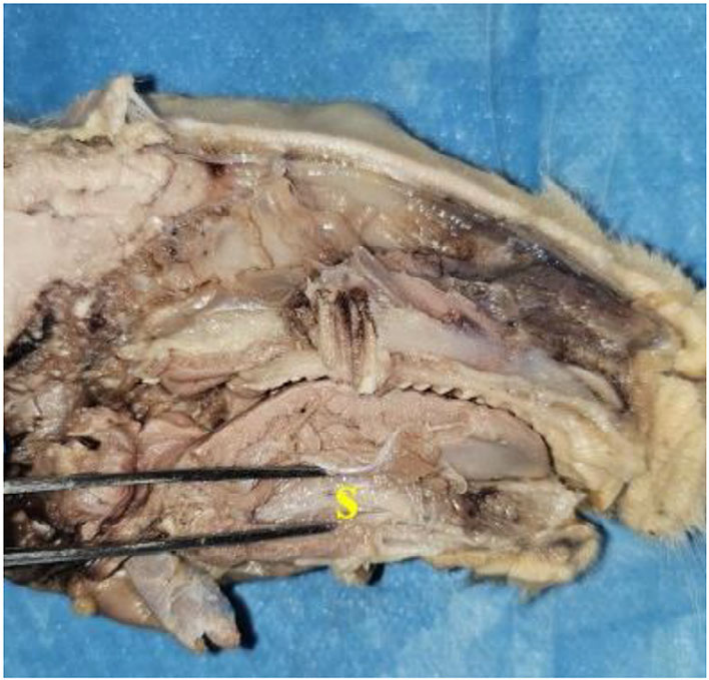
Sublingual gland in the rabbit (**s**: sublingual gland).

**FIGURE 16:**
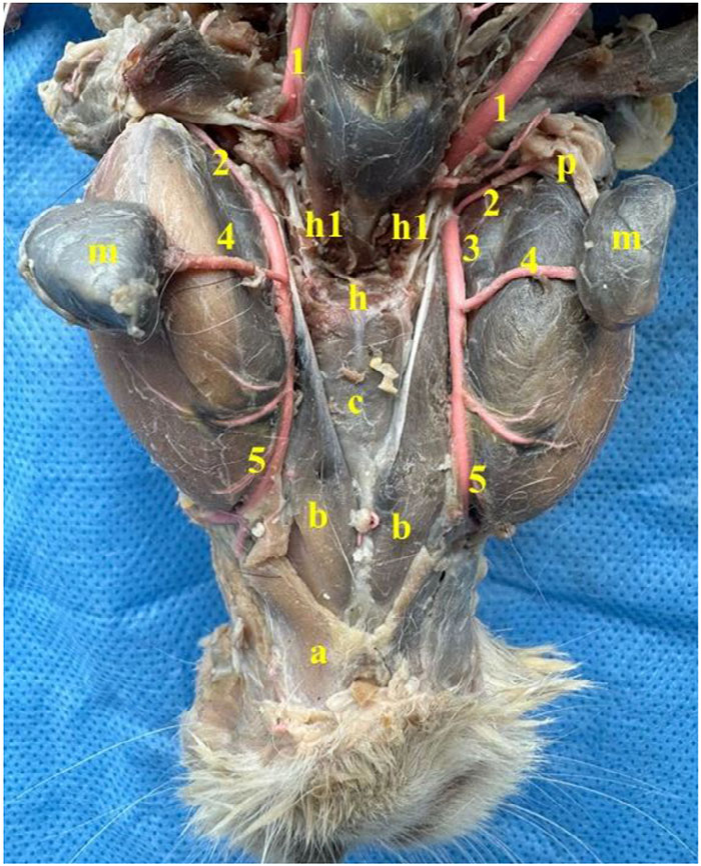
Anatomical structures seen in the Rabbit with its head dissected from the ventral side (**a**: mylohyoid muscle, **b**: digastricus muscle, **c**: geniohyoideus muscle, **h**: basihyoid of hyoid bone, **h1**: thyrohyoid of hyoid bone, **p**: ventral part of the parotid gland, **m**: mandibular gland, **1**: external carotid artery, **2**: branch of the facial artery leading to parotid gland, **3**: facial artery, **4**: branch of facial artery to mandibular gland, **5**: facial artery).

It was seen that there were five different salivary glands in the rabbit: parotid gland, zygomatic gland, mandibular gland, buccal gland, and sublingual gland. While sublingual gland was appeared to be single, the other glands were seen to be paired. Parotid gland was revealed to be brownish grey in colour on the ventral side of the ear (Figure 11). Zygomatic gland was found to be located side by side with the lacrimal gland in the anteroventral corner of the orbit (Figure 12). It was seen that it was located between the lacrimal bone and zygomatic arc, immediately caudal to the malar muscle and even partly under the malar muscle. Mandibular gland was appeared to be located ventromedial to the mandibular angle (angulus mandibula) (Figure 13). Buccal gland was found to be located caudal to the oral angle (angulus oris). It was seen to be localised between the cranial of the masseter muscle (Figure 14). It was named as buccal gland because the region it is located is bucca. It was seen that the sublingual gland was localised between the root of the tongue and frenulum of the tongue (phrenilum linguae) (Figure 15). Morphometric values ​​of the salivary glands are shown in Tables 4, 5, 6, 7, 8. When we compared, it was seen that the salivary gland with the largest length, width, and weight was the parotid gland. The gland with the smallest length and weight was the buccal gland, and the gland with the smallest width was the sublingual gland. It was seen that the first branch given by the facial artery at the level of the angular process of the lower jaw bone after it was separated from the linguofacial trunk provided vascularisation of the ventral part of the parotid gland. It was determined that the continuation of the same vessel gave its second branch at the level of the middle of the lower jaw bone's body to provide arterial vascularisation of the mandibular gland (Figure 16). It was observed that the arterial vascularisation of the zygomatic gland was vascularised by thin branches originating from the external ophthalmic artery. It was determined that the arterial vascularisation of the buccal gland was vascularised by thin branches originating from the inferior labial artery. It was appeared that the arterial vascularisation of the sublingual gland was vascularised by thin branches originating from the sublingual artery (Figure 10).

**TABLE 4:** Parameters of parotid glands (mm).

Parameters	Minimum	Maximum	Mean + standard deviation
**GPDB**	38.34	73.33	54.75±11.52
**GPDG**	32.96	55.61	45.82±7.25
**GPDA**	22.24	42.43	33.28±5.92
**GPSB**	8.36	14.64	10.78±2.13
**GPSG**	56.80	76.56	68.67±5.79
**GPSA**	44.98	70.52	55.88±8.83
**GSB**	19.44	22.10	20.66±1.04
**GSG**	4.10	4.22	4.18±0.04
**GSA**	0.29	0.34	0.32±0.02

Abbreviations: GPDB, length of right parotid gland; GPDG, width of right parotid gland; GPDA, weight of right parotid gland (g); GPSB, length of left parotid gland; GPSG, width of left parotid gland; GPSA, weight of left parotid gland (g).

**TABLE 5:** Parameters of zygomatic gland (mm).

Parameters	Minimum	Maximum	Mean + Standard deviation
**GZDB**	25.28	50.49	34.02±9.72
**GZDG**	10.09	21.09	15.92±3.25
**GZDA**	0.27	0.69	0.44±0.15
**GZSB**	19.47	29.40	22.78±3.20
**GZSG**	7.99	16.28	11.34±2.72
**GZSA**	0.21	0.55	0.39±0.12

Abbreviations: GZDB, length of right zygomatic gland; GZDG, width of right zygomatic gland; GZDA, weight of right zygomatic gland (g); GZSB, length of left zygomatic gland; GZSG, width of left zygomatic gland; GZSA, weight of left zygomatic gland (g).

**TABLE 6:** Parameters of buccal gland (mm).

Parameters	Minimum	Maximum	Mean + standard deviation
**GBDB**	12.85	17.50	14.98±1.51
**GBDG**	10.08	11.98	10.66±0.65
**GBDA**	0.16	0.48	0.31±0.12
**GBSB**	14.16	18.18	16.25±1.50
**GBSG**	7.65	9.97	8.84±0.89
**GBSA**	0.12	0.34	0.22±0.08

Abbreviations: GBDB, length of right buccal gland; GBDG, width of right buccal gland; GBDA, weight of right buccal gland (g); GBSB, length of left buccal gland; GBSG, width of left buccal gland; GBSA, weight of left buccal gland (g).

**TABLE 7:** Parameters of mandibular gland (mm).

Parameters	Minimum	Maximum	Mean + standard deviation
**GMDB**	13.68	23.49	18.01±3.36
**GMDG**	8.22	13.23	10.09±1.96
**GMDA**	0.37	1.74	0.73±0.44
**GMSB**	12.43	20.84	16.31±2.66
**GMSG**	7.76	11.72	10.06±1.35
**GMSA**	0.32	0.84	0.62±0.17

Abbreviations: GMDB, length of right mandibular gland; GMDG, width of right mandibular gland; GMDA, weight of right mandibular gland (g); GMSB, length of left mandibular gland; GMSG, weight of left mandibular gland; GMSA, weight of left mandibular gland (g).

**TABLE 8:** Parameters of sublingual gland (mm).

Parameters	Minimum	Maximum	Mean + standard deviation
**GSB**	19.44	22.10	20.66±1.04
**GSG**	4.10	4.22	4.18±0.04
**GSA**	0.29	0.34	0.32±0.02

Abbreviations: GSB, length of sublingual gland; GSG, width of sublingual gland; GSA, weight of sublingual gland (g).

**FIGURE 17:**
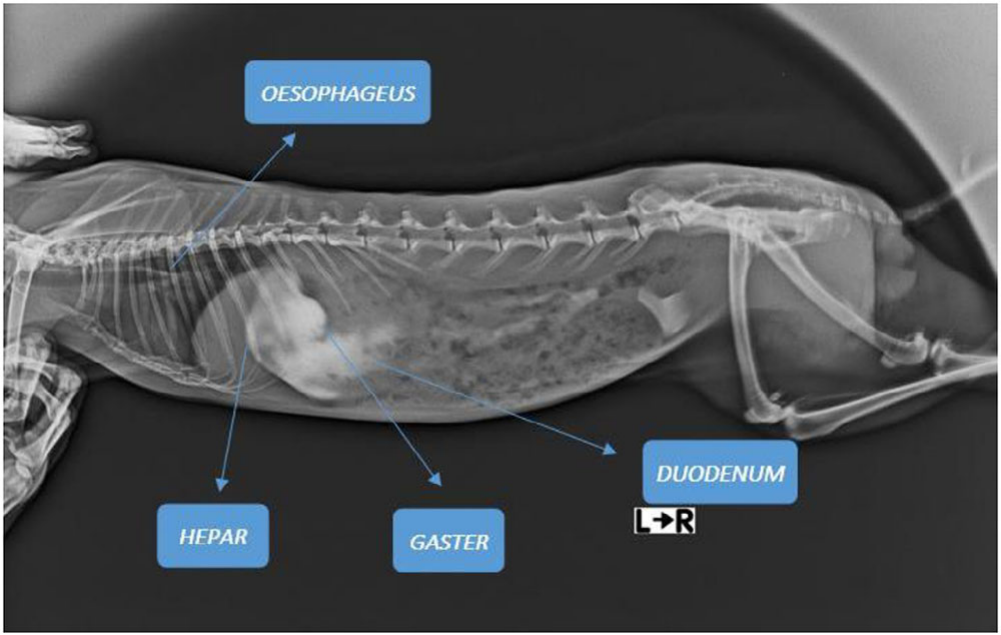
X‐ray image of the oesophagus, liver, stomach, and duodenum in the rabbit.

It was revealed that the oesophagus continued dorsal to the trachea and entered the thoracic cavity (cavum thoracis) in parallel with the trachea. The oesophagus was examined in two sections as cervical part (pars cervicalis) and thoracal part (pars thoracalis). It was seen that the third section of the oesophagus, the abdominal part (pars abdominalis), was not long enough to be measured because it opened into the stomach immediately after the diaphragm. The total oesophagus length was appeared as 15.85 mm and weight as 2.41 g. It was determined that the length of the thoracal part was twice that of the cervical part. The X‐ray image of the oesophagus and upper digestive system organs in the rabbit is shown in Figure 17. The parameters of the oesophagus are presented in Table 9.

**TABLE 9:** Parameters of oesophagus (cm).

Parameters	Minimum	Maximum	Mean + standard deviation
**OPCB**	4.423	5.937	51.04±5.52
**OPCG**	1.045	1.975	1.603±2.61
**OPTB**	7.381	12.835	10.481±18.19
**OPTG**	1.349	1.941	1.706±2.03
**OA**	1.50	3.13	2.41±0.60

Abbreviations: OPCB, length of oesophagus's pars cervicalis; OPCG, width of oesophagus's pars cervicalis; OPTB, length of oesophagus's pars thoracica; OPTG, width of oesophagus's pars thoracica; OA, weight of oesophagus (g).

## 4 Discussion

Because of their phylogenetically similarity to humans, laboratory animals such as New Zealand rabbits are frequently chosen for scientific research (Mapara et al. 2012).

The lips, which round the mouth opening, serve a variety of purposes in newborn animals, including sucking, communication, and nutrient absorption (König and Liebich 2015). The organs that contain the sensory hairs, or whiskers, that give certain animal species their extremely precise tactile sensitivity (Feldmeyera et al. 2013). Compared to normal hairs, sensory hairs have substantially deeper roots (König and Liebich 2015). Brito et al. (2021) documented the occurrence of a cleft in the rice rat, while McLaughlin and Chiasson (1979) identified a similar defect in the upper lip of the rabbit. In the current investigation, a cleft was likewise observed in the upper lip. This cleft is referred to as an upper lip cleft or harelip (Donnelly and Vella 2016). According to Covașă and Munteanu (2019), the cranial thyroid artery represents the initial branch of the common carotid artery, a finding that is consistent with the observations of the present study. Furthermore, while Covașă and Munteanu (2019) reported that the occipital and internal carotid arteries arise from a common origin, the current study found these vessels to emerge as distinct branches. Previous literature indicates that in rabbits, the facial artery courses along the anterior border of the masseter muscle, from which the inferior labial artery originates. It has also been noted that the facial artery continues as the superior labial artery, which supplies the upper lip (McLaughlin and Chiasson 1979; Özer 1991). In the current investigation, consistent with the observations of Özer (1991), the inferior labial artery was found to originate from the facial artery and supply the lower lip. It was also determined that the superior labial artery, a prominent branch continuing from the facial artery, provided vascularisation to the upper lip. The data obtained on dental formula were in agreement with the findings of Donnelly and Vella (2016). The rabbits exhibited 2 incisors in the maxilla, 1 incisor in the mandible, 3 premolars in the upper jaw, 2 premolars in the lower jaw, and 3 molars in both the upper and lower jaws. It was observed that the first and second upper incisors were comparable in size. However, our findings concerning the dimensions of incisors 1 and 2 diverged from those reported by Donnelly and Vella (2016). While the present study found both teeth to be of similar length, Donnelly and Vella (2016) described incisor 1 as being larger than incisor 2.

According to Vetscraft (2021), the hard palate in rabbits is comprised of 15 palatine rugae. However, in the present study, a total of 13 pairs of palatine rugae were identified. Among these, eight pairs were found to be situated within the diastema region. In comparison, Brito et al. (2021) noted the presence of only three pairs of palatine rugae in the diastema of the rice rat. In rabbits, the ascending palatine artery, which originates from the maxillary artery, is responsible for the arterial supply of the palate. The minor palatine artery is known to vascularise the soft palate, whereas the major palatine artery supplies the hard palate (Özer 1991). These findings are consistent with those of the current study, which observed that the ascending palatine artery arising from the maxillary artery contributes to the vascularisation of the hard palate. It was also established that both the ascending palatine and greater palatine arteries are involved in the arterial perfusion of the hard palate, while the lesser palatine artery is responsible for supplying the soft palate.

Previous studies have indicated that, in contrast to rats, rabbits possess a pair of palatine tonsils located in the oropharyngeal region (Casteleyn et al. 2011; Craigie 1969; Gültiken 2010). Nevertheless, in the present anatomical investigation, no evidence of palatine tonsils was detected through macroanatomical analysis.

According to Matosz et al. (2018), rabbits possess five distinct salivary glands: the parotid, zygomatic, mandibular, buccal, and sublingual glands. In contrast, Donnelly and Vella (2016) reported the presence of four paired glands, explicitly excluding the buccal gland. The parotid gland has been described as a diffuse, white to brownish structure (Bensley, 1991). In the present study, the parotid gland was observed as brownish‐grey in colour and located on the ventral aspect of the ear. Consistent with the findings of Matosz et al. (2018), the parotid gland was identified as the largest among the salivary glands. The zygomatic gland, also referred to as the orbital gland, has been reported to lie between the zygomatic arch and the orbit, exhibiting a lobulated morphology. In prior studies, it was described as light brown in colour and weighing approximately 0.60–0.70 g. In the present investigation, the zygomatic gland was located between the zygomatic arch and the lacrimal bone, just caudoventral to the malar muscle, consistent with previously reported anatomical landmarks. The measured weight of the gland was 0.41 g, which was lower than the value reported in the literature. Matosz et al. (2018) also stated that the mandibular gland is composed of two rounded lobes, a finding that was confirmed in this study. It has been described as a 2 cm‐long gland, weighing approximately 0.75–0.80 g, and located ventromedially to the mandibular angle. The mandibular duct is said to originate from the dorsal surface of the gland, passing dorsally to the digastricus muscle, and crossing the lingual nerve before opening adjacent to the frenulum of the tongue (Barone 2011). In the present study, the average length of the mandibular gland was found to be 17 mm, with a mean weight of 0.67 g, values that are comparable to those cited in the literature. Çalışlar (1987) noted that the buccal gland is distributed throughout the cheek region, while McLaughlin and Chiasson (1979) described it as comprising two lobes. However, macroanatomical observations in the current study revealed that the buccal gland consisted of a single, large lobe, which differs from previous reports.

Barone (2011) stated that the buccal gland is anatomically subdivided into the superior and inferior buccal glands. According to Çalışlar (1987), the sublingual gland in rabbits is located between the pars molaris of the mandible and the lingual body, whereas Kuru et al. (2017a) reported its position in blind mice as being in the ventral region of the tongue root. In the present study, however, the sublingual gland was observed between the tongue root and the frenulum, indicating a more ventral localisation compared to previous findings.

Bensley (1910) noted that the sublingual gland in rabbits is undivided, consisting of a single lobe rather than separate major and minor parts. This anatomical feature was also confirmed in our study. While Matosz et al. (2018) identified the sublingual gland as the smallest among the salivary glands, our findings revealed that the buccal gland held this distinction in the current investigation. It is hypothesised that biological variables such as age, sex, and environmental or geographic conditions may contribute to these morphological differences.

Cížek et al. (2023) reported the absence of a median sulcus on the tongue in the Patagonian rabbit, while noting the presence of a well‐developed lingual torus. The torus of the tongue has also been documented in certain myomorph rodent species (Kılınç et al. 2010; Wannaprasert 2018). In the current investigation, both the median sulcus and the lingual torus were observed in rabbits. Praag (2024) reported that the total tongue length in rabbits measured 65 mm, with a width ranging from 15 to 17 mm. In contrast, the present study recorded an average tongue length of 52.39±1.75 mm, width of 17.38±2.20 mm, and a weight of 5.82±1.13 g. When compared to Praag's findings, the tongue length in this study was shorter, while the width remained comparable. Abumandour (2014) described the presence of four distinct types of lingual papillae in rabbits: filiform, fungiform, foliate, and vallate papillae. However, Kuru et al. (2017b) reported that only filiform and vallate papillae are present in mole rats. In addition, Abumandour and El‐Bakary (2013) found that while filiform, fungiform, and vallate papillae are common in herbivorous species, foliate papillae may be absent. Notably, all four types of papillae were identified in the Patagonian rabbit (Cížek et al. 2023). The findings of the present study, based on macroanatomical examination, confirmed the presence of all four papillae on the rabbit tongue: filiform, fungiform, foliate, and vallate. Regarding vascularisation, it has been observed that in rabbits the lingual artery originates from the external carotid artery in conjunction with the facial artery, forming the linguofacial trunk (Özer 1991). Following its origin, the lingual artery passes between the genioglossus and hyoglossus muscles and continues as the deep lingual artery. These anatomical features were also evident in the present study. Furthermore, the lingual artery was observed to bifurcate at the level of the tongue root into the deep lingual and sublingual arteries.

It was observed that the oesophagus, similar to that in blind mice (İlgün 2008), originates from the dorsal aspect of the trachea and proceeds into the thoracic cavity parallel to the trachea. Timothy (1990) described the oesophagus in rabbits, and İlgün and Özkan (2012a) in blind mice, as comprising three segments: cervical, thoracic, and abdominal. In the current study, the oesophagus was examined only in two segments: the cervical and thoracic parts. The oesophageal length in rabbits has been reported as 3.62±0.97 cm in juveniles, 6.97±0.46 cm in subadults, and 8.19±0.26 cm in adults (Selim et al. 2017). Measurements in blind rats indicated an oesophagus length of 7.67 cm (İlgün 2008), while guinea pigs present lengths ranging from 8 to 11 cm (Cooper and Schiller 1975), hamsters from 10 to 12 cm (Hoffman et al., 1968), and mice from 7 to 8 cm (Çalışlar 1987). Other studies report the length from the pharynx to the stomach as approximately 13–15 cm (Barone 2011; Huffman 1958), with a diameter close to 1 cm. Nath et al. (2016) recorded the mean oesophageal length and diameter in white rabbits as 9.62±1.64 cm and 1.16±0.12 cm, respectively, with an oesophagus weight of 1.57±0.278 g. In contrast, the present study found a total oesophageal length of 15.85 cm and a weight of 2.41 g. Additionally, it was determined that the thoracic segment was approximately twice the length of the cervical segment.

## 5 Conclusions

In conclusion, the macroanatomical structure of the upper digestive system organs in the rabbit, morphometric values, ​​and the arteries supplying these organs were determined by detailed dissections. In addition to the obtained morphometric values, it is thought that the presented study will contribute to the anatomy literature by determining the vertebral projections of the upper digestive system organs and the small vascular variations. It is also anticipated that the study will help clinicians and veterinarians by revealing the location of the upper digestive system organs and their relationship with neighbouring organs through imaging methods such as X‐ray.

## Author Contributions

**Gülseren Kirbaş Doğan** and **Elif Duman Çabakçor**: Conceptualisation, Data curation, Formal analysis, Funding acquisition, Investigation, Methodology, Project administration, Resources, Software, Supervision, Validation, Visualisation, Writing – original draft, Writing – review & editing

## Ethic Statement

Necessary ethics committee approvals were obtained.

## Conflicts of Interest

There is no conflict of interest for the authors. This study has not been submitted to any other journal.

## Peer Review

The peer review history for this article is available at https://www.webofscience.com/api/gateway/wos/peer‐review/10.1002/vms3.70505.

## Data Availability

The data that support the findings of this study are available from the corresponding author upon reasonable request.

## References

[vms370505-bib-0001] Abumandour, M. M. A. 2014. “Morphological Comparison of the Filiform Papillae of New Zealand White Rabbits (*Oryctolagus cuniculus*) as Domestic Mammals and Egyptian Fruit Bat (*Rousettus aegyptiacus*) as Wild Mammals Using Scanning Electron Microscopic Specimens.” International Journal of Morphology 32, no. 4: 1407–1417.

[vms370505-bib-0002] Aycan, K. , and A. Bilge . 1984. “Plastik Enjeksiyon Ve Korozyon Metodu ile Vaskuler Sistemin anatomisinin araştırılması.” Ege Üniversitesi Tıp Fakültesi Dergisi 6, no. 4: 545–552.

[vms370505-bib-0003] Ayırtır Başdinç, Y. 2023. “Balb‐c Ve Swiss Albino Irkı Farelerin Gastrointestinal Sisteminin karşılaştırmalı Morfometrik Analizi.” *Master Thesis*, Van Yüzüncü Yıl Üniversitesi.

[vms370505-bib-0004] Barone, R. 2011. Artères. Anatomie Comparée Des Mammifères Domestiques, Tome 5: Angiologie. 2nd ed, 103–448. Editions Vigot.

[vms370505-bib-0005] Bensley, B. A. 1910. Practical Anatomy of the Rabbit: An Elementary Laboratory Textbook in Mammalian Anatomy. P. Blakiston's Son & Co.

[vms370505-bib-0006] Brito, J. , N. Tinoco , J. Curay , and U. F. J. Pardiñas . 2021. “New Morphological Data on the Rare Sigmodontine *Mindomys hammondi (Rodentia, Cricetidae*), an Arboreal Oryzomyine From North‐Western Andean Montane Forests.” Neotrop Biol Conserv 16, no. 3: 397–410.

[vms370505-bib-0007] Bugge, J. 1963. “A Standardised Plastic Injection Technique for Anatomical Pusposes.” Acta Anatomica 51: 177–192.

[vms370505-bib-0008] Çalışlar, T. 1987. *Laboratuvar Hayvanları Anatomisi*. İstanbul Üniversitesi Yayınları.

[vms370505-bib-0009] Casteleyn, C. , S. Breugelmans , P. Simoens , and W. V. Broeck . 2011. “The Tonsils Revisited: Review of the Anatomical Localization and Histological Characteristics of the Tonsils of Domestic and Laboratory Animals.” Clinical & Developmental Immunology 2011: 472460. 21869895 10.1155/2011/472460 PMC3159307

[vms370505-bib-0010] Cížek, P. , K. Go ´zdziewska‐Harłajczuk , P. Hamouzová , J. Kle ´ckowska‐Nawrot , and P. Kvapil . 2023. “Lingual Ultrastructuraland Histochemical Study in the Patagonian mara (Rodentia: Caviidae, *Dolichotis patagonum*) in Relation to Other Hystricomorphs.” Animals 13: 3889. 38136926 10.3390/ani13243889 PMC10740948

[vms370505-bib-0011] Cooper, G. , and A. L. Schiller . 1975. Anatomy of the Guinea Pig. Harvard University Press.

[vms370505-bib-0012] Cotozzolo, E. , P. Cremonesi , G. Curone , et al. 2021. “Characterization of Bacterial Microbiota Composition Along the Gastrointestinal Tract in Rabbits.” Animals 11: 31. 10.3390/ani11010031 PMC7824689 33375259

[vms370505-bib-0013] Covașă, C. T. , and A. C. Munteanu . 2019. “Angioarchitectonics and Distribution of Common Carotid Artery Terminals in Domestic Rabbit.” Bulletin of University of Agricultural Sciences and Veterinary Medicine Cluj‐Napoca Veterinary Medicine 76: 20.

[vms370505-bib-0014] Craigie, E. H. 1969. Bensley's Practical Anatomy of the Rabbit. 8th ed. University of Toronto Press.

[vms370505-bib-0015] Davies, R. R. , and J. A. E. Davies . 2003. “Rabbit Gastrointestinal Physiology.” The Veterinary Clinics of North America. Exotic Animal Practice 6: 139–153. 12616837 10.1016/s1094-9194(02)00024-5

[vms370505-bib-0016] Demiraslan, Y. , and M. O. Dayan . 2021. *Veteriner Sistematik Anatomi*. Nobel Kitabevi.

[vms370505-bib-0017] Donnelly, T. M. , and D. Vella . 2016. “Anatomy, Physiology and Non‐Dental Disorders of the Mouth of Pet Rabbits.” Veterinary Clinics of North America: Exotic Animal Practice 19: 737–756. 10.1016/j.cvex.2016.04.004. 27497204

[vms370505-bib-0018] Dursun, N. 2012. Veteriner Anatomi II, 139–160. Medisan Yayınevi.

[vms370505-bib-0019] Dyce, K. M. , V. O. Sack , and C. J. G. Wensing . 2010. Textbook of Veterinary Anatomy. 5th ed. Elsevier.

[vms370505-bib-0020] Erençin, Z. , O. Hassa , M. Sağlam , and A. Evren . 1967. “Enjeksiyon Yolu Ile Damar Ve Kanal Sistemleri Için plastik demonstrasyon Metodlarının geliştirilmesi.” Ankara Üniversitesi Veteriner Fakültesi Dergisi 14, no. 3: 444–452.

[vms370505-bib-0021] Feldmeyera, D. , M. Brechtd , F. Helmchene , et al. 2013. “Barrel Cortex Function.” Progress in Neurobiology 103: 3–27. 23195880 10.1016/j.pneurobio.2012.11.002

[vms370505-bib-0022] Greene, E. C. 1963. Anatomy of Rat, 283. Hafner Publishing Company.

[vms370505-bib-0023] Gültiken, M. E. 2010. “Deney Hayvanları Anatomisi.” In Yayın Yeri: Ondokuz Mayıs Üniversitesi Yayınları, edited by A. Aksoy , F. Kolbakır , and M. Hökelek , 1–37. Basım Sayısı .

[vms370505-bib-0024] Hoffman, R. A. , P. F. Robinson , and H. Magalhaes . 1968. The Golden Hamsters Biology and Use in Medical Research. The Iowa Univ. Press.

[vms370505-bib-0025] Holmes, D. D. 1988. Clinical Labaratory Animal Medicine. Lowa: The Lowa, 100–115. State University Press.

[vms370505-bib-0026] Huffman, K. W. 1958. “Gross and Histological Studies on the Digestive Tract of the Rabbit.” Master thesis, *Oklahoma State University of Agriculture and Applied Science*.

[vms370505-bib-0027] Ilgün, R. 2008. Körfarelerde (Spalax leucodon) Canalis Alimentarius, Hepar Ve Pancreas’ ın, Makro Ve Mikro Anatomisi Üzeride Incelemeler. PhD thesis, Fırat University Institute of Health Sciences, Elazığ.

[vms370505-bib-0028] Ilgün, R. , and Z. E. Ozkan . 2012a. “Körfarelerde (*Spalax leucodon*) Canalis Alimentarius Makroanatomisi Üzerinde Incelemeler.” Atatürk Üniversitesi Vet Bil Derg 7, no. 2: 85–91.

[vms370505-bib-0029] Kılınç, M. , S. Erdogan , S. Ketani , and M. A. Ketani . 2010. “Morphological Study by Scanning Electron Microscopy of the Lingual Papillae inthe Middle East Blind Mole Rat (*Spalax ehrenbergi, Nehring, 1898*).” Anatomia, Histologia, Embryologia 39: 509–515. 20649897 10.1111/j.1439-0264.2010.01022.x

[vms370505-bib-0030] Killman, K. 2009. “4‐H Rabbit Manual.” Ministry of Agriculture and Lands.

[vms370505-bib-0031] König, H. E. , and H. G. Liebich . 2015. Veterinary Anatomy (*Domestic Mammals*). Medipres, Malatya.

[vms370505-bib-0032] Kuru, N. , K. Çinar , E. Demirbag , and R. Ilgün . 2017a. “Histological and Histochemical Structure of Lingual Salivary Glands in Mole Rat (*Spalax leucodon*).” Indian Journal of Animal Research 51, no. 2: 252–255.

[vms370505-bib-0033] Kuru, N. , K. Çinar , E. Demirbag , and R. Ilgün . 2017b. “Some Lectin Binding Properties of the Tongue of the Mole Rat, *Nannospalax Xanthodon* .” Biotechnic & Histochemistry 92, no. 6: 445–449. 28862488 10.1080/10520295.2017.1341057

[vms370505-bib-0034] Malewitz, T. D. 1965. “Normal Histology of the Digestive Tract of the Mouse.” Okajimas Folia Anatomica Japonica 41: 21–47. 14325623 10.2535/ofaj1936.41.1_21

[vms370505-bib-0035] Mapara, M. , B. S. Thomas , and K. M. Bhat . 2012. “Rabbit as an Animal Model for Experimental Research.” Dental Research Journal 9, no. 1: 9–111. 10.4103/1735-3327.92960 PMC3283968 22363373

[vms370505-bib-0036] Matosz, B. , C. Dezdrobitu , C. O. Martonoș , V. Luca , and A. Damian . 2018. “Topography of the Major Salivary Glands in Rabbits.” Scientific Works. Series C. Veterinary Medicine LXIII, no. 2:.

[vms370505-bib-0037] McLaughlin, C. A. , and R. B. Chiasson . 1979. Laboratory Anatomy of the Rabbit, 46–51. Wm. Brown Company Publishers.

[vms370505-bib-0038] Mukhopadhyay, S. , and L. R. Wagner . 2020. Rabbıt Anatomy: A Brief Photographıc Atlas and Dıssection Guide. 2th ed, Augusta University.

[vms370505-bib-0039] N.A.V . 2017. “International Committee on Veterinary Gross Anatomical Nomenclature. Nomina Anatomica Veterinaria (NAV).” 6th ed, World Association of Veterinary Anatomists, Hanover (Germany), Ghent (Belgium), Columbia, MO (U.S.A.), Rio de Janeiro (Brazil).

[vms370505-bib-0040] Nath, S. K. , S. Das , J. Kar , K. Afrin , A. K. Dash , and S. Akter . 2016. “Topographical and Biometrical Anatomy of the Digestive Tract of White New Zealand Rabbit (*Oryctolagus cuniculus*).” Journal of Advanced Veterinary and Animal Research 3, no. 2: 145–151.

[vms370505-bib-0041] Özer, M. 1991. Yerli Kedi Ve Beyaz Yeni Zelanda Tavşanının arteria carotis communis'i Üzerinde Komparatif makroanatomik araştırmalar. PhD thesis, Ankara University Institute of Health Sciences.

[vms370505-bib-0042] Perpiñán, D. 2019. “Rabbit Neutering.” Companion Animal.

[vms370505-bib-0043] Potter, G. E. , E. L. Rabb , L. W. Gibbs , and A. B. Medlen . 1956. “Anatomy of the Digestive System of Guinea Pig (*Cavia porcellus*).” Bios 27, no. 4: 232–234.

[vms370505-bib-0044] Praag, E. V. 2024. “Anatomy of the Tongue in Rabbits.” *MediRabbit.com*.

[vms370505-bib-0045] Reece, W. O. 2015. Dukes' Physiology of Domestic Animals, 13th ed. Wiley.

[vms370505-bib-0046] Selim, A. , E. Hazaa , and W. Goda . 2017. “Comparative Histological Studies of the Esophagus Wall of Oryctolagus Cuniculus Rabbit Adult, Young and Lactating Using Light Microscope.” Journal of Cytology and Histology 8: 2.

[vms370505-bib-0047] Timothy, P. J. 1990. “Comparative Gross Anatomical Studies of the Rabbit Digestive System.” *DVM* ABU, Student Project.

[vms370505-bib-0048] Vdoviaková, K. , E. Petrovová , M. Maloveská , et al. 2016. “Surgical Anatomy of the Gastrointestinal Tract and Its Vasculature in the Laboratory Rat.” Gastroenterology Research and Practice 2016: 2632368. 26819602 10.1155/2016/2632368 PMC4706906

[vms370505-bib-0049] Vetscraft . 2021. “Hard Palate (Palatum durum) of Animals.” Vetscraft.

[vms370505-bib-0050] Wannaprasert, T. 2018. “Morphological Characteristics of the Tongue and Lingual Papillae of the Large Bamboo Rat (*Rhizomys sumatrensis*).” Anatomical Science International 93: 323–331. 28952063 10.1007/s12565-017-0414-x

